# Major Membrane Protein TDE2508 Regulates Adhesive Potency in *Treponema denticola*

**DOI:** 10.1371/journal.pone.0089051

**Published:** 2014-02-21

**Authors:** Yuki Abiko, Keiji Nagano, Yasuo Yoshida, Fuminobu Yoshimura

**Affiliations:** Department of Microbiology, School of Dentistry, Aichi Gakuin University, Kusumoto-cho, Chikusa-ku, Nagoya, Aichi, Japan; University of Oklahoma Health Sciences Center, United States of America

## Abstract

The cultivation and genetic manipulation of *Treponema denticola*, a Gram-negative oral spirochaeta associated with periodontal diseases, is still challenging. In this study, we formulated a simple medium based on a commercially available one, and established a transformation method with high efficiency. We then analyzed proteins in a membrane fraction in *T*. *denticola* and identified 16 major membrane-associated proteins, and characterized one of them, TDE2508, whose biological function was not yet known. Although this protein, which exhibited a complex conformation, was presumably localized in the outer membrane, we did not find conclusive evidence that it was exposed on the cell surface. Intriguingly, a TDE2508-deficient mutant exhibited significantly increased biofilm formation and adherent activity on human gingival epithelial cells. However, the protein deficiency did not alter autoaggregation, coaggregation with *Porphyromonas gingivalis*, hemagglutination, cell surface hydrophobicity, motility, or expression of Msp which was reported to be an adherent molecule in this bacteria. In conclusion, the major membrane protein TDE2508 regulates biofilm formation and the adhesive potency of *T*. *denticola*, although the underlying mechanism remains unclear.

## Introduction

*Treponema denticola* is a Gram-negative anaerobe that is classified as a spirochaete and has periplasmic flagella, which confer motility to enable the bacterium to move in a semisolid medium [1]. The bacterium is a member of the “red complex” bacteria, which are critical pathogens associated with human periodontal diseases [2], and is also believed to influence arteriosclerosis [3]. *T*. *denticola* colonizes and forms a biofilm in the gingival sulcus, further exacerbating inflammation and destruction of periodontal tissues [4].

The virulence factors of *T*. *denticola* have been reported and are summarized in reviews [1], [5], [6]. Msp (named from major sheath protein), the most abundant protein in the bacteria, acts as an adherent factor to bacteria and host tissues [7], [8]. It has also reported to function as a porin [9], [10]. Although the localization of Msp has been argued [11]–[13], Anand *et al*. recently demonstrated that it was localized in the outer membrane and exposed on the surface [10]. However, a substantial quantity of Msp also exists in the periplasm [10], [11]. The chymotrypsin-like protease dentilisin is also a major virulence molecule in this pathogen [14], [15]. Dentilisin is a complex consisting of several proteins, and affects various host functions, such as activation of the complement system and degradation of cytokines and other host proteins 1,5,6. It is also reported that dentilisin functions as an adherent molecule to other oral bacteria [16] and host molecules [17]. Although other molecules involving in pathogenicity have been also reported, little is known about the pathogenic mechanisms of *T*. *denticola*, largely because of the difficulties in handling this organism; complicated media such as TYGVS and NOS are usually required for its culture [18]. Additionally, genetic manipulation of *T*. *denticola*, such as the construction of genetic mutants, is still challenging [19]. In this study, we found that *T. denticola* grew well in a medium that was formulated based on a commercially-available medium, and we also established a highly efficient method for genetic modification.

Bacterial surface molecules are important for growth and pathogenicity because they directly interact with environmental factors such as other bacteria and host tissues [1]. They often play a critical role especially in biofilm formation and adhesion to host cells. *T*. *denticola* has an outer membrane at the outermost layer, but its composition is totally different from a general outer membrane of Gram-negative bacteria. The outer membrane of *T*. *denticola* does not contain lipopolysaccharide; rather, it has a lipid that is similar to lipoteichoic acid found in Gram-positive bacteria [12], [20]. Although *T*. *denticola* has a unique outer membrane, few studies have conducted a comprehensive investigation of its surface molecules [21], [22]. In this study, we analyzed the major membrane-associated proteins of *T*. *denticola*, identified an unknown protein TDE2508, and demonstrated that this protein regulated biofilm formation and adherence to host cells.

## Materials and Methods

### *T*. *denticola* Strains and Culture Conditions

We primarily used *T*. *denticola* ATCC 35405, and also used ATCC 33520 strain, which were provided by the RIKEN BRC through the National Bio-Resource Project of the MEXT, Japan. For the bacterial culture, we largely used Modified GAM (Nissui Pharmaceutical Co., Ltd., Tokyo, Japan) supplemented with 0.001% thiamine pyrophosphate and 5% heat-inactivated rabbit serum (herein referred to as mGAM-TS). We also used two additional media; TYGVS, which is widely used for the culture of *T*. *denticola* [18], and Modified NOS (mNOS), which is a relatively simple medium [23]. The bacteria were anaerobically and statically cultivated at 37°C. When needed, highly pure agar (Difco Agar Noble, Becton, Dickinson and Company, Franklin Lakes, NJ, USA) and antibiotics (described in detail below) were added to the media. For the osmotic pressure test, NaCl and KCl were added. *T*. *denticola* was generally cultivated in mGAM-TS until the late logarithmic phase for use in the experiments.

### Antibiotics and Antibiotic Sensitivity Test

For the selection of transgenic mutants and antibiotic sensitivity testing, we used the following antibiotics: ampicillin, chloramphenicol, erythromycin, gentamicin, kanamycin, penicillin G, tetracycline, and vancomycin (all were obtained from Sigma-Aldrich, St. Louis, MO, USA). The minimum inhibitory concentration (MIC) was evaluated by employing the liquid dilution assay. Briefly, bacterial culture was inoculated in mGAM-TS broth at 0.1 of an optical density (OD) at 620 nm (OD620). After 5 days of anaerobic incubation, the turbidity was measured at 620 nm and the growth was determined.

### Subcellular Fractionation

Subcellular fractionation was performed as described previously [24]. All procedures were performed under cold conditions. *T*. *denticola* cells were washed in a buffer consisting of 20 mM Tris, pH 7.5, supplemented with protease inhibitors (1 mM phenylmethylsulfonyl fluoride, 0.1 mM *N*-α-*p*-tosyl-L-lysine chloromethyl ketone and 0.1 mM leupeptin). The cells were disrupted in a French pressure cell by passing them three times at 100 MPa in the presence of 25 µg/mL DNase and RNase. The undisrupted cells were removed by centrifugation at 1,000×*g* for 10 min. The resultant whole cell lysate (WCL) was subjected to ultracentrifugation at 100,000×*g* for 60 min. The supernatant and sediment were collected as soluble and envelope fractions, respectively. For further fractionation of the envelope fraction, it was suspended in a buffer containing 0.5–8% Triton X-100. The soluble and insoluble fractions in the Triton X-100-containing buffer were separated by ultracentrifugation at 100,000×*g* for 60 min. The protein concentration was determined using a Pierce BCA Protein Assay kit (Thermo Scientific, Rockford, IL, USA).

We also extracted a surface layer from intact cells of *T*. *denticola* in a similar manner as described previously [25]. Briefly, washed bacterial cells were gently suspended and incubated for 5 min at room temperature in phosphate-buffered saline (PBS), pH 7.4, supplemented with 0.1% Triton X-100, then centrifuged at 4,000×*g* for 15 min. The supernatant was filtrated with a 0.22-µm pore filter membrane and concentrated by ammonium sulfate precipitation. After dialysis, it was subjected to SDS-PAGE and Western blot analyses as described below. The remaining cell pellet was observed by electron microscopy to confirm disappearance of the cell surface layer and existence of the cell body.

### SDS-PAGE and Western Blot Analyses

The samples were denatured in a buffer containing 1% SDS with 0.4 M 2-mercaptoethanol (2-ME) at 100°C for 5 min, unless otherwise noted. SDS-PAGE gels were stained with Coomassie brilliant blue R-250 (CBB). The protein concentration was estimated by comparison with the protein bands obtained for a known quantity of bovine serum albumin. For Western blotting, the protein bands in the gel were electrophoretically transferred to a PVDF membrane. The membrane was blocked with 5% skim milk in Tris-buffered saline (TBS), pH 7.5, with 0.05% Tween 20. Subsequently, the membrane was incubated with specific antisera as described below, followed by incubation with peroxidase-conjugated anti-rabbit IgG. Amersham ECL Prime Western Blotting detection reagent (GE Healthcare UK Limited, Buckinghamshire, UK) was used for development of the target bands.

### Mass Spectrometry Analysis

CBB-stained protein bands were identified by MALDI-TOF MS [26]. After in-gel tryptic digestion, the peptides were extracted, desalted, and analyzed using a 4800 MALDI TOF/TOF Analyzer (Life Technologies Corporation, Carlsbad, CA, USA). The identity of the proteins was deduced from the MS peaks by a comparative analysis of the mass with that in the Mascot database (http://www.matrixscience.com/). Annotation and homology searches were also performed using BLAST (http://blast.ncbi.nlm.nih.gov/Blast.cgi).

### Preparation of Antisera

The *tde2508* (denotes gene name in this paper) gene encoding the entire TDE2508 (denotes protein name) protein was amplified by PCR from the chromosomal DNA of ATCC 35405 using primers His-2508-F and 2508-R (Table 1). To add a hexahistidine tag, the His-2508-F primer included a DNA sequence encoding an amino acid sequence of MGSSHHHHHHSSG. The DNA fragment was cloned in a vector pCR-Blunt II-TOPO (Life Technologies Corporation) and the integrity of the nucleotides was confirmed. Although we tried to purify the His-tagged TDE2508 using a Ni-affinity column, our attempts were unsuccessful since the recombinant protein formed an insoluble inclusion body. Therefore, we purified the protein by extracting the corresponding band after separation by SDS-PAGE. The purified protein was confirmed to be TDE2508 by mass spectrometry. Anti-TDE2508 antiserum was obtained by immunizing a rabbit with the purified protein emulsified with Freund’s complete adjuvant. We also prepared an antiserum to whole cells of *T*. *denticola* by immunizing a rabbit with whole cells of the bacteria. We confirmed that the antiserum to the whole cells significantly reacted to Msp and TmpC in this organism (data not shown). This study was carried out in strict accordance with the recommendations of the Regulations on Animal Experimentation at Aichi Gakuin University. The protocol was approved by the University Animal Research Committee (permit number: AGUD 065). All surgery was performed under sodium pentobarbital anesthesia, and all efforts were made to minimize animal suffering.

**Table 1:** Primers used in this study.

Primer	Symbol in Fig. 1 *	Sequence (5′ to 3′)	Description
His-2508-F	a	ATGGGCAGCAGCCATCATCATCATCATCACAGCAGCGGCCAAATATCGATGACATCATATAGTACTC	Forward primer to amplify *tde2508* linked with the DNA sequence encoding the amino acid sequence of MGSSHHHHHHSSG indicated by underline
2508-R	b	TTAGAATTTAACCGATAGGGCAAATT	Reverse primer to amplify *tde2508*
2508U-F	c	AAACCTTGAAGAATTCGTAAACTTGTG	Forward primer to amplify upstream region of *tde2508*
2508U-R	d	GAAGGATGAAATTTTTCAGGGACAACTTGATATCTCCTTTTTAAAATCTACTATAGCCGATTATC	Reverse primer to amplify upstream region of *tde2508*, fused with initial region of *ermB* indicated by underline
2508D-F	e	CTATGAGTCGCTTTTGTAAATTTGGAAAGACTATTAAAATCTACTTTAGATAAAACCTGTTAATGTTTAC	Forward primer to amplify downstream region of *tde2508*, fused with terminal region of *ermB* indicated by underline
2508D-R	f	AAATATGAGTAAGGGCTTTCTCCG	Reverse primer to amplify downstream region of *tde2508*
*ermB*-F	-	AAGTTGTCCCTGAAAAATTTCATCCTTC	Forward primer to amplify *ermB* including the promoter region
*ermB*-R	-	CTTTCCAAATTTACAAAAGCGACTCATAG	Reverse primer to amplify *ermB* including the promoter region
2509-F	g	ACTGCTTTCAAGGGCTTCT	Forward primer to anneal within *tde2509*
2509-R	h	AAATATGAGTAAGGGCTTTCTCCG	Reverse primer to anneal within *tde2509*

**Figure 1:**
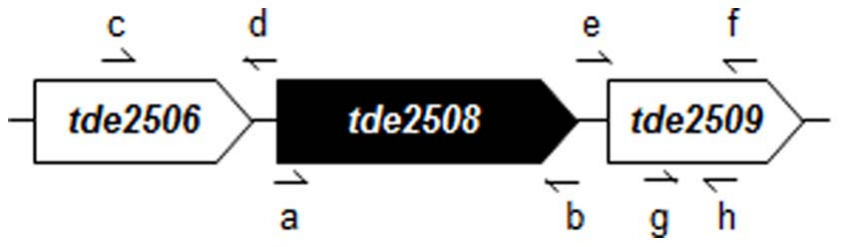
Gene map of *tde2508* and its neighbors on the chromosomal DNA of *T*. *denticola* ATCC 35405. The small arrows show primers which are presented in Table 1. The *tde2508* and *tde2509* genes were co-transcribed (data not shown).

* Symbols are shown in Fig. 1.

### Construction of a *tde2508*-deletion Mutant

We prepared electrocompetent cells of *T*. *denticola* as follows. *T*. *denticola* culture was spread on mGAM-TS solidified with 2.5% agar and cultivated anaerobically at 37°C for 1 week. The cells were then collected from the surface of the agar plate with a cotton swab and suspended in chilled 1 mM HEPES, pH 7.4. The cells were washed three times with the HEPES buffer and once with 10% glycerol by centrifuging at 4,000×*g* for 10 min at 4°C, and finally suspended in 10% glycerol. The cell concentration was adjusted so that the OD600 was 10, which corresponded to 5×10^10^ cells/mL. The competent cells were prepared immediately prior to use to avoid freeze-thaw of the cells.

The *tde2508* gene was deleted by replacing it with an erythromycin-resistant gene (*ermB*) [27]. Briefly, a *tde2508*-deletion cassette was constructed using a PCR-based overlap-extension method. The relevant primers are shown in Fig. 1 and Table 1. The 824-bp upstream region (ending immediately before the translation initiation codon; primers 2508U-F/2508U-R) and 839-bp downstream region (beginning immediately after the terminating nonsense codon; primers 2508D-F/2508D-R) of the *tde2508* gene were amplified by PCR from the chromosomal DNA of ATCC 35405, and the 1036-bp region containing the *ermB* gene (primers *ermB*-F/*ermB*-R) was amplified from pVA2198 [28]. These 3 amplicons were fused into 1 piece by the overlap extension method [29] using primers 2508U-F and 2508D-R. The final 2,699-nucleotide fragment was cloned into a pGEM-T Easy vector (Promega, Madison, WI, USA), and sequenced to confirm that there were no errors introduced during PCR amplification.

The plasmid construct (50 µg) was linearized by digestion with restriction enzymes, purified, and dissolved in 50 µL of TE buffer. The linearized plasmid (50 µL) and 50 µL of the competent cells were set together in an electroporation cuvette with a 0.2-cm gap, then pulsed at 1.8 kV for approximately 5 ms. The pulsed cells were immediately transferred into 2 mL of mGAM-TS that was warmed under anaerobic conditions beforehand, and anaerobically incubated at 37°C for at least 24 h. Semisolid mGAM-TS containing 0.8% agar supplemented with 40 µg/mL erythromycin was used for the selection of transformants. Semisolid media were often used for the isolation of *T*. *denticola* clones [30] because the bacteria hardly form a colony from single cells on a solid agar medium. The selection medium was melted and incubated at 40°C to maintain its condition. The culture, including the transformants, was gently mixed in the selection medium, poured into a dish and anaerobically incubated at 37°C for 7 days.

### Analysis of Transcriptional Activity

To investigate polar effects by the mutation of *tde2508*, we examined the transcriptional activity of *tde2509*, which is a downstream gene of *tde2508* (Fig. 1). Total RNA was isolated using ISOGEN (Nippon Gene Co., Ltd, Tokyo, Japan) and treated with RNase-free recombinant DNase I (Takara Bio Inc., Otsu, Japan) to eliminate contaminating genomic DNA. The purified RNA (100 ng) was used to generate cDNA with a PrimeScript RT-PCR Kit (Takara Bio Inc.). The resulting cDNA was used as a template for PCR. The primers 2509-F and 2509-R are used to examine the transcription of *tde2509* (Fig. 1 and Table 1). After a standard 10, 15, 20, and 25-cycle PCR, the transcriptional activity was determined by analyzing the target band after agarose electrophoresis and ethidium bromide-staining of the gel.

### Chemical Crosslinking Assay

The chemical crosslinking assay was performed as described previously [31]. *T*. *denticola* cells were suspended in PBS, pH 7.4. The cross-linker suberic acid bis *N*-hydroxysuccinimide ester, spacer arm length 11Å (Sigma-Aldrich) was added at a final concentration of 10–200 mM. After incubation of the reaction mixture at 4°C for 2 h, the crosslinking reaction was stopped by the addition of 1.0 M Tris buffer, pH 8.0. The reacted cells were disrupted by sonication and analyzed by Western blotting.

### Slide Agglutination Assay

The slide agglutination assay was performed following a standard protocol. *T. denticola* ells were washed twice with PBS, pH 7.4, and the OD600 was adjusted to 0.5. The bacterial cells were mixed with the antisera to whole cells of *T*. *denticola* and TDE2508.

### Immunofluorescence Assay

*T. denticola* culture was applied to a well of the filtration plate (MultiScreen-GV, 96-well membrane plate, 0.22 µm pore size, Millipore Corporation, Billerica, MA, USA). The solutions in the wells were removed through the membrane by centrifugation at 2,000×*g* for 3 min. The wells were blocked with TBS containing 3% bovine serum albumin at room temperature for 15 min and were incubated with antisera to whole cells of *T*. *denticola* or TDE2508 (1∶1,000 dilutions) for 30 min at room temperature. After washing three times with TBS, Alexa Fluor 488-conjugated goat IgG fraction to the rabbit IgG secondary antibody (1∶1,000 dilution; Life Technologies Corporation) was added and incubated for 30 min at room temperature in the dark. After washing, the cells were suspended in a small volume of TBS, placed on the slide glass and mounted using ProLong Gold antifade reagent (Life Technologies Corporation). The stained cells were examined by confocal laser scanning microscopy (LSM 710, Carl Zeiss, Oberkochen, Germany).

### Biotinylation of Cell Surface Proteins

Cell surface labeling with bulky biotin reagent was performed as described previously [32]. *T*. *denticola* cells suspended in PBS, pH 8.0, and supplemented with 1 mM MgCl_2_ were labeled with EZ-Link NHS-PEG12-Biotin (Thermo Scientific) at 4°C for 30 min. The reaction was stopped by the addition of 0.1 M glycine. The biotinylated cells were disrupted by sonication, subjected to SDS-PAGE and blotted onto a nitrocellulose membrane. Biotinylation was detected using peroxidase-conjugated streptavidin (Dako, Glosrup, Denmark) and Amersham ECL Prime Western Blotting detection reagent.

### Biofilm Assay

The turbidity of the *T*. *denticola* cells in a fresh medium was adjusted so that the OD600 was 0.2. Aliquots (10 mL) were poured into a 60-mm dish (Iwaki, a brand of Asahi Glass Co. Ltd., Tokyo, Japan) and then anaerobically incubated at 37°C for 48 h. We also used plates coated with human collagen type I and human fibronectin (Iwaki). Unbound bacterial cells were removed by gently washing with PBS, pH 7.4, and the cells were then collected in 0.2 mL of PBS, pH 7.4, by scraping. The biofilm volume was evaluated by measuring the cell suspension at OD600. The biofilm was also investigated by scanning electron microscopy (SEM) as described below.

### Assay of Adherence to Gingival Epithelial Cells

Human gingival epithelial cells, Ca9-22 (provided by the RIKEN BRC), were seeded into 8-well Lab-Tek II chamber slides (Thermo Scientific) and cultivated in DMEM with 10% fetal bovine serum, heat-inactivated under 5% CO_2_ at 37°C for 24 h to near confluence at 5 ×10^5^ cells per well. *T*. *denticola* cells were washed, and the OD600 of the cell concentration in DMEM with 10% fetal bovine serum was adjusted to 0.1 and 1.0 (corresponding to 5×10^8^ and 5×10^9^ cells/mL, respectively). Then 0.4 mL of each bacterial suspension was added to a well of the slide, corresponding to multiplicity of infection (MOI) values of 400 and 4,000, respectively. After incubation under 5% CO_2_ at 37°C for 1 h, the chamber slides were gently washed three times with PBS, pH 7.4, and the cells were fixed with 4% paraformaldehyde in PBS. The fixed cells were washed three times with PBS and then permeabilized with PBS containing 0.1% Triton X-100 at 37°C for 30 min. The cells were washed again and then blocked with TBS containing 3% bovine serum albumin at room temperature for 30 min. The slides were incubated with anti-*T*. *denticola* whole cell antiserum (1∶1,000 dilution) for 30 min at room temperature and washed three times with TBS. Then, Alexa Fluor 488-conjugated goat IgG fraction to the rabbit IgG secondary antibody (1∶1,000 dilution) and Alexa Fluor 568-conjugated phalloidin (1 µg/mL; Life Technologies Corporation) were simultaneously added and incubated for 60 min at room temperature in the dark, in order to label bacterial cells and actin filaments of Ca9-22 cells, respectively. After extensive washing with TBS, the chamber slides were mounted using ProLong Gold antifade reagent. The stained slides were examined by confocal laser scanning microscopy. *T*. *denticola* cells that adhered to the epithelia were counted in the captured images, in which a field corresponded to 0.045 mm^2^.

### Autoaggregation, Coaggregation and Hemagglutination Assays

For all assays, *T. denticola* cells were washed twice with 20 mM phosphate buffer, pH 8.0 supplemented with 1 mM CaCl_2_, 1 mM MgCl_2_, and 150 mM NaCl, and the OD600 was adjusted to 0.5. For the autoaggregation assay, 1 mL of the cell suspension was placed in a cuvette and the OD600 values were intermittently monitored. For the coaggregation assay, we used another red complex bacteria, *Porphyromonas gingivalis* ATCC 33277. *P*. *gingivalis* was cultivated in mGAM (without any supplements) and prepared in the manner described for *T*. *denticola*. Each bacterial suspension was mixed in an equal volume and the OD600 values were monitored. The hemagglutination assay was performed in a microtiter plate as described previously [33]. Human (O-type), rabbit and chicken red blood cells were washed in PBS, pH 7.4. Bacterial cells were mixed with an equal volume of a 1% red blood cell suspension, incubated at 4°C for 1 h, and the hemagglutination was determined.

### Cell Surface Hydrophobicity Assay

The hydrophobicity assay was performed as described previously [15]. Briefly, the washed cells were suspended in a phosphate buffer containing 0.03 M urea and 0.8 mM MgCl_2_, and the OD400 was adjusted to 0.5. Aliquots (1.2 mL) in tubes were vigorously mixed with *n*-hexadecane (0.6 mL) for 60 s. The OD400 of the aqueous phase was measured as an index of hydrophobicity. The relative hydrophobicity of the cell surface was calculated using the following formula: % hydrophobicity = [1- (OD400 after mixing)] ×100/(OD400 before mixing).

### Motility

The OD600 of *T*. *denticola* cells in mGAM-TS was adjusted to 0.2. The cell suspensions (1 µL) were carefully placed on mGAM-TS agar plate which was solidified by adding agar at 0.3% and 0.5% final concentrations. The plate was anaerobically incubated at 37°C, and the turbid plaque was monitored as an index of bacterial motility. *T*. *denticola* does not spread on an agar medium, but does penetrate into the agar medium.

### Electron Microscopy

*T*. *denticola* cells were negatively stained with 1% ammonium molybdate, pH 7.0. Whole cell samples were observed and photographed using a JEM-1210 transmission electron microscope (TEM, JEOL, Tokyo, Japan). We also observed *T*. *denticola* by SEM to investigate the biofilm. *T. denticola* cells were incubated on a cover glass as described for the biofilm assay. Then, the cells were fixed and dehydrated following a standard method. Cells were coated with 5 nm of platinum and observed using a scanning electron microscope (JXA-8530FA, JEOL).

### Statistical Analysis

The data were evaluated using Student’s *t* tests. Statistical differences were considered significant at *p*<0.05. All experiments were repeated at least three times except for those presented in Fig. S3 in the supporting information.

## Results and Discussion

### Cultivation of *T*. *denticola* in a Novel Medium

For the cultivation of *T*. *denticola*, complicated media such as TYGVS and NOS are widely used [18]. However, Ruby *et al*. showed that *T*. *denticola* grew in a relatively simple medium consisting of brain-heart infusion broth and some supplements, which called Modified NOS (mNOS, which is entirely different from NOS) [23]. This prompted us to find an alternative simple medium, such as one based on a commercially available medium. To this end, we tried Modified GAM (mGAM), which is widely used in Japan as a general medium for cultivation of anaerobic bacteria. We added 0.001% thiamin pyrophosphate (an active form of thiamin) because *T*. *denticola* has a deficiency in thiamin synthesis [34]–[36]. Additionally, heat-inactivated rabbit serum was added to the media, since addition of 10% serum was generally necessary for *T. denticola* cultivation. The addition of 5% and 10% sera in mGAM showed a similar growth of *T. denticola*, while the addition of 2.5% decreased the growth (data not shown). Therefore, our final medium comprised mGAM supplemented with 0.001% thiamin pyrophosphate and 5% rabbit serum, and was named mGAM-TS.

**Figure 2:**
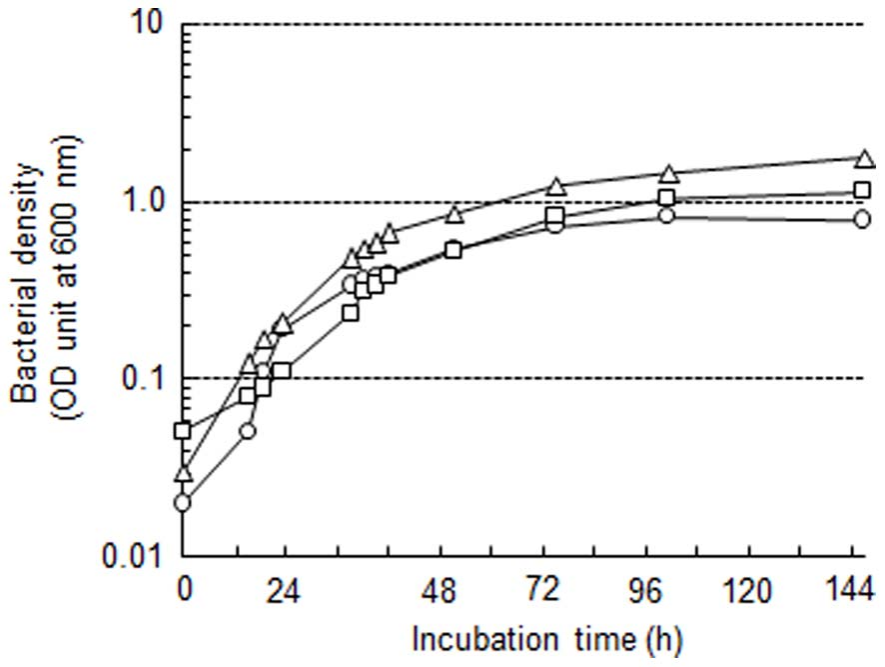
Growth curves of *T*. *denticola*. *T*. *denticola* ATCC 35405 was inoculated in mGAM-TS (5% serum, circle), TYGVS (10% serum, triangle), and mNOS (10% serum, square), and anaerobically incubated at 37°C. The optical density (OD) at 600 nm was monitored. The figure shows a representative image from one of three independent experiments.

We compared the growth of *T*. *denticola* in mGAM-TS (5% serum), TYGVS (10% serum), and mNOS (10% serum) (Fig. 2). The mGAM-TS provided a similar rate of growth compared to the other media although the plateau was slightly lower for mGAM-TS. After the inoculation, the bacteria showed a logarithmic growth between 24–48 h, and then reached a stationary phase. We decided to use a 48-h culture (late logarithmic phase) for the other experiments in this study.

The use of complicated media hampers the study of *T*. *denticola*. Therefore, mGAM-TS, which is based on a commercially available medium and easily prepared, offers an advantage to the study of this organism. Another strain of *T*. *denticola* ATCC 35020 showed similar growth (data not shown), and mGAM-TS was also useful as selection medium for transformants (described below). These indicates the versatility of this medium.

### Analysis of Major Membrane-Associated Proteins in *T*. *denticola*

**Figure 3:**
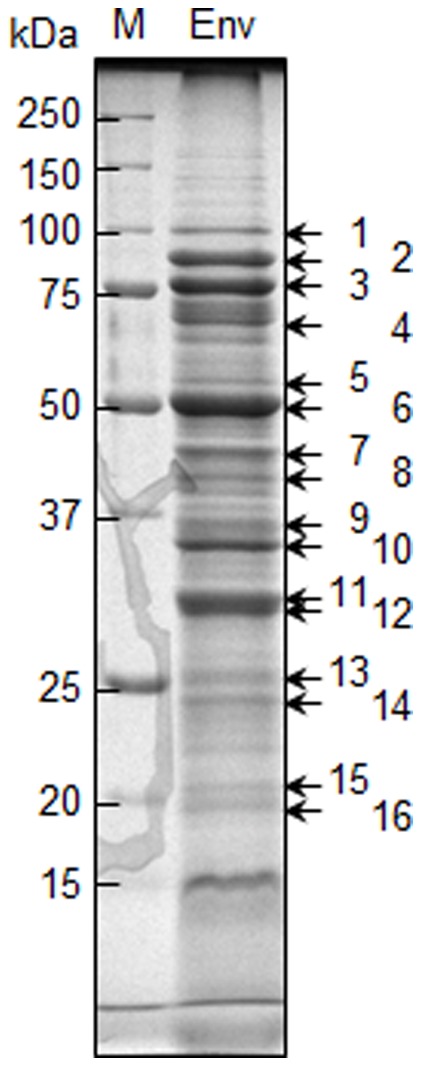
Major membrane proteins on an SDS-PAGE gel. Ten micrograms of the envelope fraction (Env) of *T*. *denticola* ATCC 35405 was separated by SDS-PAGE and stained with CBB. The numbers (1–16) indicate proteins identified by mass spectrometry. Annotation of each protein is shown in Table 2. M denotes a standard marker.

We analyzed the major proteins of the envelope (membrane) fraction in *T*. *denticola* ATCC 35405. Ten micrograms of the fraction was subjected to SDS-PAGE, then stained with CBB. The major proteins (i.e., intense bands containing more than 100 ng of protein or 1% of total protein) were analyzed by mass spectrometry, and 16 proteins were identified (Fig. 3 and Table 2). Flagellar proteins (#10, 11, 12 and 14) were detected in the envelope fraction although the flagella reside in the periplasmic space in the bacteria. In addition, cytoplasmic filament protein A (#3) was detected although it exists in cytosol as a cytoskeletal protein [37]. However, it is reasonable that these were fractionated into the envelope fraction because they anchor to the inner membrane [37]. Indeed, flagella of this bacteria were reported to be fractionated into the envelope fraction [21]. Among the uncharacterized proteins including bands #7, 13, and 15, we chose to characterize the most intense band (#7, TDE2508) in this study.

**Table 2:** Major proteins of the envelope fraction (including of the inner and outer membranes) in *T*. *denticola*.

Band #	Gene ID*	Description
1	0844	pyruvate phosphate dikinase
2	1072	putative lipoprotein, probably OppA associated protein
3	0842	cytoplasmic filament protein A, CfpA
4	1071	peptide ABC transporter, peptide-binding protein OppA
5	1273	oligopeptide/dipeptide ABC transporter, peptide-binding protein
6	0405	major outer sheath protein (Msp)
7	2508	hypothetical protein
8	2056	outer membrane hemin-binding protein A
9	1950	membrane lipoprotein TmpC, putative
10	1712	flagellar filament outer layer protein, FlaA
11	1004	flagellar filament core protein
12	1475	flagellar filament core protein
13	1584	putative lipoprotein
14	1408	flagellar filament outer layer protein FlaA, putative
15	0731	hypothetical protein
16	0955	LemA family protein

*The prefix of TDE is abbreviated from Gene ID.

TDE2508 consists of 455 amino acids, and the molecular weight was calculated to be 50708.47 Da. BLAST search showed that TDE2508 exists in other *T*. *denticola* strains including ATCC 33520, MYR-T, H1-T, AL-2, ASLM, US-Trep, F0402, and H-22, and there are also homologous proteins in *T*. *socranskii* and *T*. *azotonutricium*, but not in *T. pallidum*. SignalP (http://www.cbs.dtu.dk/services/SignalP/) predicted that 22 amino acids in the N-terminal region present a signal peptide. The predicted signal sequence was consistent with the characteristics of the well-characterized signal peptides of *T*. *denticola* and other spirochaetes [38]. These suggest that TDE2508 is localized in the membrane or periplasm. Although we applied to N-terminal amino acid sequence analysis, it was not determined likely because of N-terminal modification of the protein. Other online programs for subcellular localization prediction, such as SOSUI (http://bp.nuap.nagoya-u.ac.jp/sosui/) and PSORT (http://www.psort.org/), also predicted TDE2508 to be a membrane protein. Phyre, which is a protein structure prediction program (http://www.sbg.bio.ic.ac.uk/~phyre/), predicted it to be a porin, which generally exists as major outer membrane proteins in Gram-negative bacteria and functions as a permeable pores to substances such as nutrients and antibiotics [39].

### Subcellular Localization and Complex Formation of TDE2508

**Figure 4:**
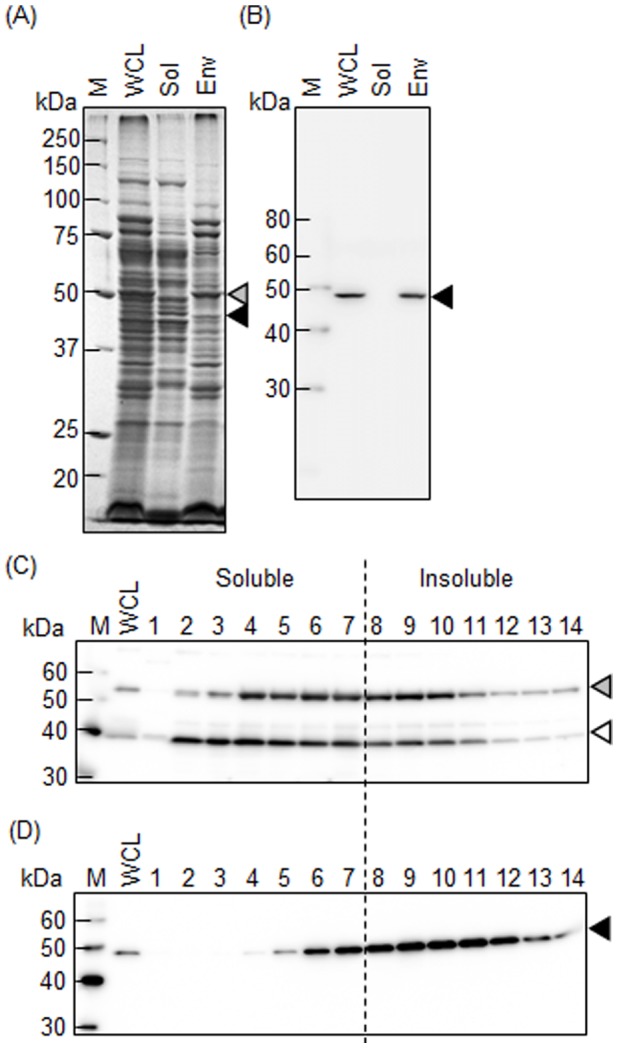
Subcellular localization of TDE2508. The whole cell lysate (WCL), soluble fraction (Sol) and envelope fraction (Env) of *T*. *denticola* ATCC 35405 were separated by SDS-PAGE with CBB-staining (A) and analyzed by Western blotting with anti-TDE2508 antiserum (B). The envelope fraction was further fractionated by differential solubilization in 0, 0.25, 0.5, 1, 2, 4, and 8% Triton X-100, and the soluble fractions (lanes 1–7, respectively) and insoluble fractions (lanes 8–14, respectively) were subjected to Western blot analysis with anti-*T*. *denticola* whole cell antiserum (C) and anti-TDE2508 antiserum (D). The grey, white and black arrowheads denote Msp, TmpC and TDE2508, respectively. M denotes a standard marker.

We examined the subcellular localization of TDE2508. TDE2508 was detected in the envelope fraction, but not in the soluble fraction (Figs. 4A and B). The envelope fraction was further fractionated by differential solubilization in Triton X-100. Fig. 4C indicated that Msp was substantially dissolved in 1% Triton X-100, suggesting that the outer membrane of *T*. *denticola* was solubilized in 1% Triton X-100 because Msp is localized in the outer membrane [10], [13]. However, TDE2508 was not sufficiently solubilized even in 4% Triton X-100, and was eventually solubilized in 8% Triton X-100 (Fig. 4D). Although these results seem to indicate that TDE2508 firmly localizes in the outer membrane, the reason for the lower solubility of TDE2508 is still unclear.

Furthermore, we found TDE2508 in the surface layer fraction extracted from intact cells of *T*. *denticola* by treating with 0.1% Triton X-100. TEM observation showed that the extraction caused loss of the most outer surface layer (i.e., outer membrane), whereas the cytoplasmic membrane (or cell body) remained (Fig. S1 in supporting information), indicating that the only surface layer was extracted. TDE2508 was detected in the surface layer fraction in SDS-PAGE and Western blot analyses (Fig. S2 in supporting information). This result supports that TDE2508 is primarily localized in the outer membrane.

Next, we examined whether TDE2508 was exposed on the cell surface. The slide agglutination assay showed that anti-TDE2508 antiserum did not cause an agglutination of intact *T*. *denticola* cells, while anti-*T*. *denticola* whole cell antiserum caused it (positive control). Furthermore, anti-TDE2508 antiserum did not label the intact cells in immunofluorescence assay, while anti-*T*. *denticola* whole cell antiserum developed an intense signal (data not shown). We also tried to label the cell surface with biotin, but the band corresponding to TDE2508 was not biotinylated (data not shown). Thus, we did not obtain any evidence that TDE2508 was exposed on the cell surface. Further studies are required to define the cellular localization by other methods such as morphological analysis.

**Figure 5:**
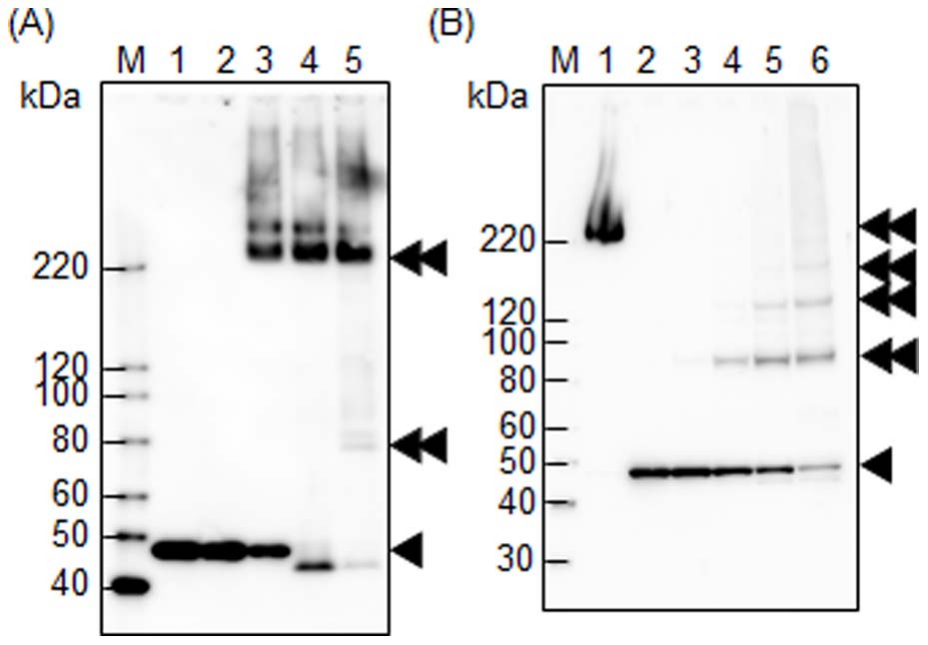
TDE2508 complex formation. (A) The whole cell lysate of *T*. *denticola* ATCC 35405 was subjected to Western blot analysis with anti-TDE2508 antiserum. Samples in lanes 1–5 were denatured by heating at 100, 80, 60, 37, and 24°C, respectively for 10 min. (B) *T. denticola* intact cells in lanes 1–6 were treated with 0, 0, 10, 50, 100, and 200 mM of a chemical cross-linker, respectively. The treated cells were sonicated and denatured by heating at 60°C for lane 1 or 100°C for lanes 2–6 for 10 min, and subjected to Western blot analysis with anti-TDE2508 antiserum. The single and double black arrowheads denote a monomer and a complex form of TDE2508, respectively. M denotes a standard marker.

We next examined TDE2508 complex formation. As the denaturation temperature decreased, the higher molecular weight bands of TDE2508 were more intense in Western blot analysis (Fig. 5A). The 2-ME present in the sample buffer did not affect the denaturation, and TDE2508 does not contain any cysteine residues. Treatment with a chemical cross-linker increased the higher molecular weight bands of TDE2508 in a dose-dependent manner (Fig. 5B). These results indicate that TDE2508 forms a complex, but it is unclear whether the complex consists of a homo- or hetero-multimer.

### Construction of a *tde2508*-deletion Mutant

We first tried to generate a *tde2508* gene-deletion mutant according to a method reported in a review by Kuramitsu *et al*. [40]. Briefly, competent cells of *T*. *denticola* were prepared from liquid culture media such as mGAM-TS as well as TYGVS, and then DNA constructs were introduced as described in the review. We used several genetic markers of antibiotic resistance including the erythromycin-resistant genes *ermF-ermB* [40] and *ermB* [27], modified gentamicin-resistant gene (*aacCm*) [41], kanamycin-resistant gene (*kanA*) [42] and chrolamphenicol-resistant gene (*cat*) [42], which was used for the selection of a genetic mutants of *T*. *denticola* or other relevant bacteria. However, we could not obtain any transformants using this method.

**Figure 6:**
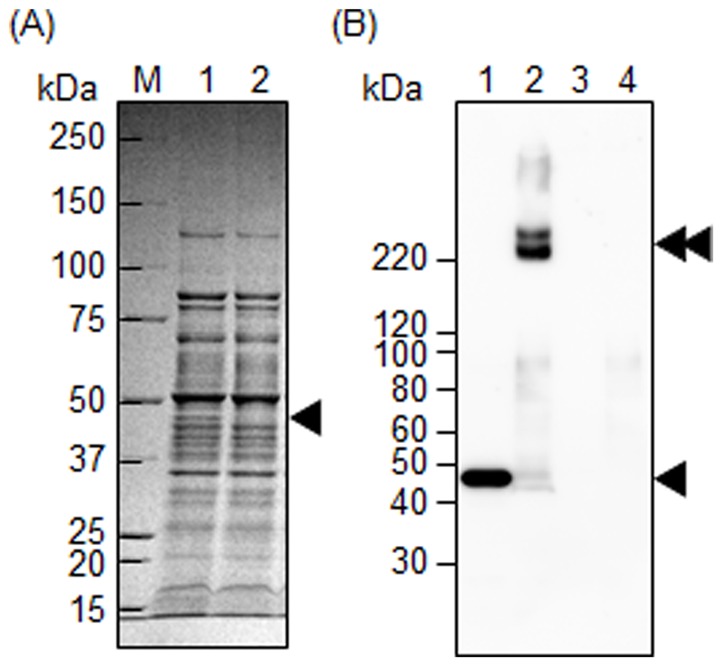
Confirmation of TDE2508 abolition in the *tde2508*-deletion mutant. The whole cell lysates of *T*. *denticola* ATCC 35405 (lanes 1 and 1–2 in panels A and B, respectively) and *tde2508*-deletion mutant (lanes 2 and 3–4 in panels A and B, respectively) were subjected to SDS-PAGE with CBB-staining (A) and Western blot analysis with anti-TDE2508 antiserum (B). Samples were denatured by heating at 100°C for 10 min, except for lanes 2 and 4 in panel B; these two were treated without heating. The single and double black arrowheads denote a monomer and a complex forms of TDE2508, respectively. M denotes a standard marker.

We have previously reported the successful transformation of *Tannerella forthysia*, an oral pathogenic bacterium, by electroporation [33]. In that study, although we could not obtain a genetic mutant by a standard protocol, we achieved the construction of a mutant when we prepared electrocompetent cells from bacterial cells grown on an agar medium. We think that collecting bacterial cells from the surface of a solid medium could reduce unfavorable ingredients and facilitate transformation. Therefore, we used the same protocol to prepare competent cells from bacterial cells developed on the surface of a solid medium in the present study. *T*. *denticola* grew on the surface of a solid medium containing 2.5% agar. When we used the competent cells and a DNA construct replaced with the *ermB* gene, colony spots of potential transformants appeared. Successful gene replacement was confirmed in all of clones tested by PCR (data not shown). We also confirmed abolition of the TDE2508 protein in one of the transformants as shown in Fig. 6; the band corresponding toTDE2508 disappeared in the *tde2508*-deletion mutant in SDS-PAGE (Fig. 6A) and Western blot analyses (Fig. 6B). Additionally, the high molecular weight bands were seen only in the wild type when the samples were denatured without heating, demonstrating that the bands were derived from TDE2508 but not artificial.

Additionally, the mutant did not show a down regulation in the transcription of *tde2509*, which is a downstream gene of and is co-transcribed with *tde2508* (data not shown).

We have constructed other mutants using this same method (unpublished data), indicating that this method is widely useful.

### General Characterization of the TDE2508-deficient Mutant

We examined the growth of the wild type and mutant of *T*. *denticola*, and observed that the mutant did not show any growth retardation in mGAM-TS (Fig. S3 in supporting information). The mutant also showed similar growth against high osmotic pressures, although the additions of NaCl and KCl decreased the growth of both strains (Fig. S3). TEM observation showed no obvious differences between the strains (Fig. S1A and B in supporting information), however, *T*. *denticola* produced a large number of vesicles (Fig. S1A and B and reference [43]), and therefore it is difficult to comment on the integrity of the outer membrane. Furthermore, we did not find an obvious difference in protein patterns between the strains (Fig. S4A).

Since TDE2508 was predicted to be a porin as described above, and since porin deficiency sometimes influences antibiotic resistance [44], we examined the MIC of several classes of antibiotics. However, the wild type and mutant showed the same MIC as follows; penicillin G, 0.013 µg/mL; ampicillin, 0.025 µg/mL; gentamicin, 0.78 µg/mL; kanamycin, 12.5 µg/mL; chloramphenicol, 25 µg/mL; tetracycline, 0.39 µg/mL; metronidazole, 10 µg/mL; vancomycin, 3.13 µg/mL. Although anaerobic bacteria are generally resistant to aminoglycoside antibiotics such as gentamicin and kanamycin, these MICs of *T*. *denticola* were considerably low.

We also examined motility, which is one of the characteristic features of *T*. *denticola*. When the bacterial cultures were placed on the semisolid media containing 0.3% and 0.5% agar, the bacteria grew by diffusely penetrating into the media. The mutant showed a similar diffusion rate to the wild type, indicating that TDE2508 did not influence the motility (Fig. S5 in supporting information).

### Adhesive Activity and Cell Surface Properties

**Figure 7:**
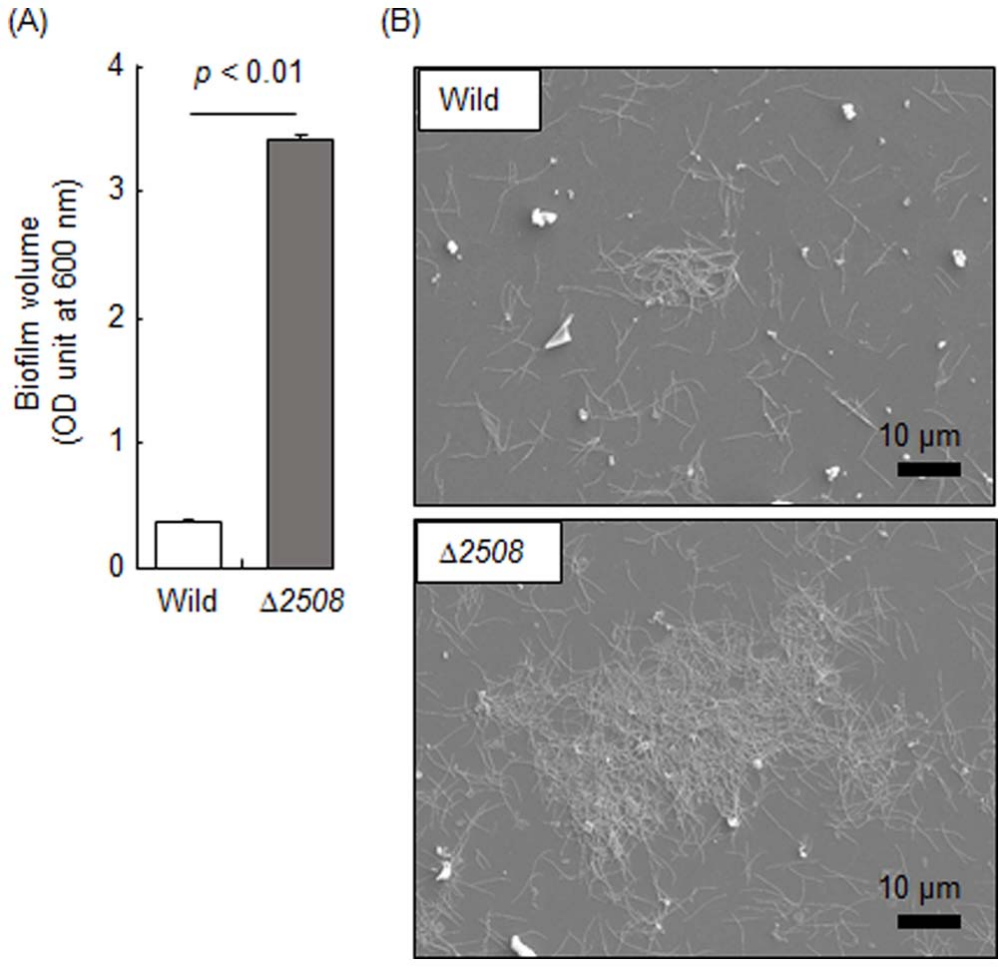
Biofilm formation. *T*. *denticola* ATCC 35405 (Wild) and *tde2508*-deletion mutant (Δ*2508*) were incubated on a polystyrene plate for 2 days. After washing, the adherent cells were collected and quantified using the optical density (OD) at 600 nm (A). Data represent means and standard deviations of two experiments performed in triplicate. Additionally, the adherent cells were observed by scanning electron microscopy (B).

**Figure 8:**
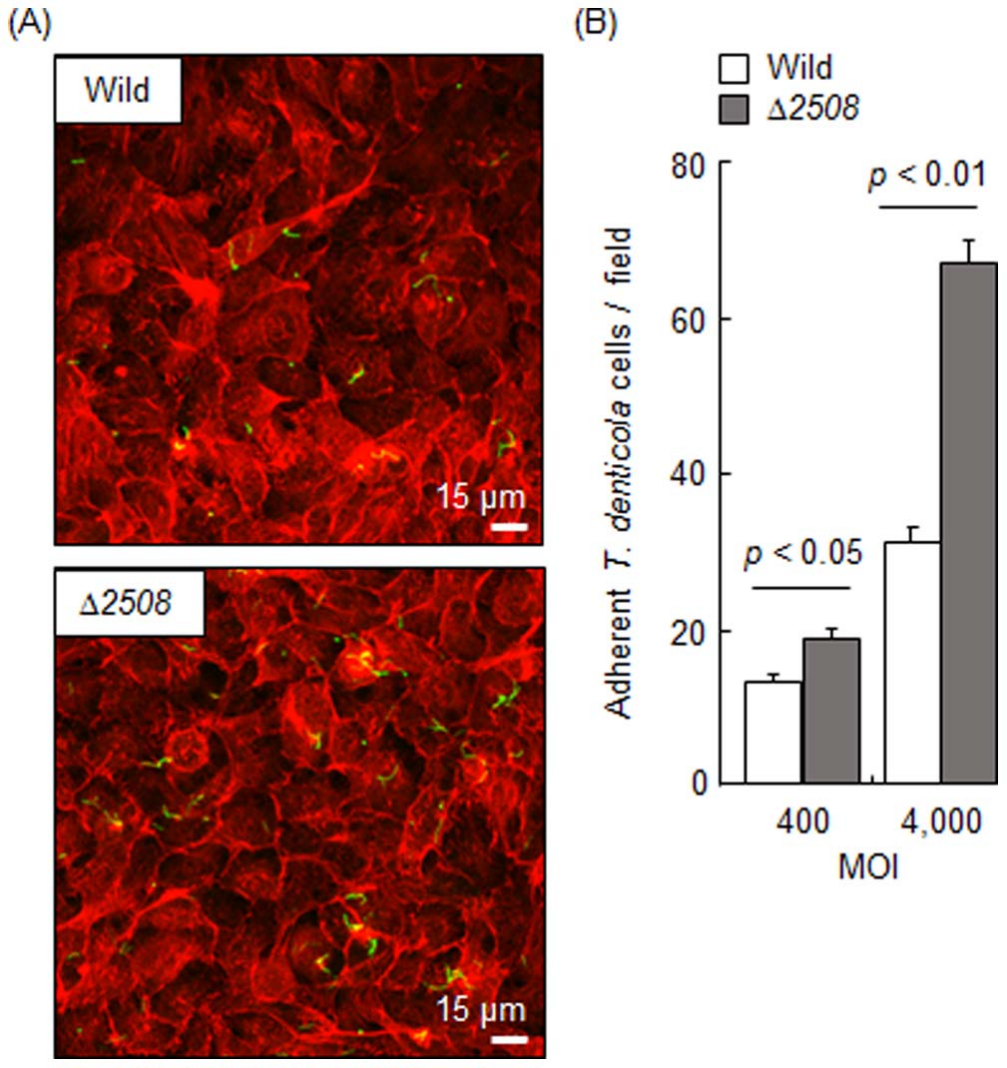
Adherence to human gingival epithelial cells. *T. denticola* ATCC 35405 (Wild) and *tde2508*-deletion mutant (Δ*2508*) were incubated with Ca9-22 epithelial cells for 1 h at 400 and 4,000 MOI. After immunostaining, the cells were observed by the confocal laser scanning microscopy (A). *T. denticola* cells and actin of the epithelial cells are shown as green and red, respectively. Each field corresponds to 0.045 mm^2^. Panel (A) shows the adherence at 4,000 MOI. We performed experiments independently twice in duplicate and counted the number of adherent *T*. *denticola* cells in 10 fields. Panel (B) shows the means and standard deviations of the number.

Although we did not obtain any evidence to indicate that TDE2508 is exposed on the cell surface, the protein likely localized in the outer membrane. Therefore we thought it might influence cell surface properties including adhesive activity. We first examined biofilm formation on a polystyrene plate. Surprisingly, the gene deletion remarkably increased biofilm formation (Fig. 7A). SEM observation confirmed that the mutant showed significantly more developed bacterial aggregates (Fig. 7B). We next examined the adherence to human gingival epithelial cells. The mutant also showed significantly higher numbers of adherent bacteria in both low (400) and high (4,000) MOI (Fig. 8A and B). In order to investigate the host ligands, we examined the bacterial adherence to human collagen type I and fibronectin which are expressed on the epithelial cells and are often reported to interact with bacterial adhesins. It was reported that *T*. *denticola* bound to fibronectin through Msp [7] and other molecules [45], and to collagens through Msp [46], [47] and other proteins [48]. However, in our hands, no substantial adherence was observed in both the wild type and mutant, and we could not compare them with a reliable model (data not shown).

We next examined the aggregation activity and hydrophobicity of the cells. Both the wild type and mutant showed only a slight autoaggregation over 6 h (Fig. S6 in supporting information). It has been reported that *T*. *denticola* co-aggregates with *P*. *gingivalis* [16], [49]. We confirmed the coaggregation with *P*. *gingivalis*, but no significant difference between the strains was observed (Fig. S6). *T*. *denticola* induces the hemagglutination of red blood cells in humans and other animals [50]. In this study, *T*. *denticola* indeed showed a hemagglutination of human, rabbit, and chicken red blood cells, but there was no difference between the strains (data not shown). Cell surface hydrophobicity was also similar between the stains; the wild type, 45.7±3.2%; *tde2508*-deletion mutant, 46.4±3.9%. We also examined the expression and subcellular localization of Msp which is reported to be an adherent factor of the bacteria [7], [8]. However, the expression and localization of Msp was similar between the strains (Fig. S4).

### Conclusions

We developed a simple medium for the cultivation of *T*. *denticola*, and improved a method for the construction of a genetic mutant. Our findings suggest that the preparation of competent cells is a critical factor in the genetic manipulation of *T*. *denticola*. We also showed that a major membrane protein, TDE2508, formed a complex and regulated biofilm formation and adhesion to epithelia, although the underlying mechanism needs to be clarified. Taking that TDE2508 was not likely exposed on the cell surface, it may be involved in processing or modifying other adherent factors.

## Supporting Information

Figure S1 **Transmission electron micrographs of** ***T*** **.** ***denticola*** **ATCC 35405 (Wild, A) and** ***tde2508*** **-deletion mutant (Δ** ***2508*** **, B).** (C) *T*. *denticola* cells treated with 0.1% Triton X-100, showing the disappearance of the surface layer. The bacterial cells were negatively stained with 1% ammonium molybdate, pH 7.0. Bars indicate 100 nm.
(TIF)

Figure S2 **Detection of TDE2508 in surface layer extraction.** Cell surface layer was extracted from intact cells of *T*. *denticola* ATCC 35405 by suspending in 0.1% Triton X-100. The extraction was subjected to SDS-PAGE with CBB-staining (A) and Western blot analysis with anti-TDE2508 antiserum (B). Lanes 1 and 2 in panel B are the whole cell lysate and the surface layer extract, respectively. The single black arrowheads denote a monomer form of TDE2508.
(TIF)

Figure S3 **Growth curves of** ***T*** **.** ***denticola*** **ATCC 35405 (Wild, A and C) and** ***tde2508*** **-deletion mutant (Δ** ***2508*** **, B and D).** The mGAM-TS medium was supplemented with NaCl (A and B) and KCl (C and D) at 0–300 mM. The strains were anaerobically incubated at 37°C and the optical density (OD) at 600 nm was monitored. The figures representative ones from two independent experiments.
(TIF)

Figure S4 **Msp expression.** Whole cell lysates of *T*. *denticola* ATCC 35405 (Wild) and *tde2508*-deletion mutant (Δ*2508*) were fractionated into soluble and envelope fractions. The envelope fractions were further fractionated by differential solubilization in 1% Triton X-100 into soluble and insoluble fractions. The samples were denatured by heating at 100°C for 10 min and subjected to SDS-PAGE with CBB-staining (A) and Western blot analysis with anti-*T*. *denticola* whole cell antiserum (B). The odd and even lanes denote Wild and Δ*2508*, respectively. Lanes 1–2, 3–4, and 5–6 are the whole cell lysate, soluble, and envelope fractions, respectively. Lanes 7–8, and 9–10 are soluble and insoluble fractions in 1% Triton X-100, respectively. The black, grey and white arrowheads denote TDE2508, Msp and TmpC, respectively. M denotes a standard marker.
(TIF)

Figure S5 **Motility test.** *T*. *denticola* ATCC 35405 (Wild) and *tde2508*-deletion mutant (Δ*2508*) were seeded on mGAM-TS agar plates which were solidified with 0.3% (left) and 0.5% (right) agar. The plates were anaerobically incubated at 37°C, and the turbid plaque was monitored for 2 weeks as an index of bacterial motility. The two strains showed almost the same motility in any concentration of agar over the entire period. Images of the plates at 7 (upper) and 14 (lower) days after the incubation are presented. The numbers in the rulers indicate centimeters.
(TIF)

Figure S6 **Aggregation assay.** For the autoaggregation test, *T*. *denticola* ATCC 35405 (open circle) and *tde2508*-deletion mutant (open triangle) were set in a cuvette, and the optical density at 600 nm (OD600) was monitored. For the coaggregation test, *T*. *denticola* ATCC 35405 (closed circle) and *tde2508*-deletion mutant (closed triangle) were mixed with *P*. *gingivalis*, and the OD600 was monitored. The open squares show autoaggregation of *P*. *gingivalis*.
(TIF)
