# Supplementary figures and images for: Major Membrane Protein TDE2508 Regulates Adhesive Potency in Treponema denticola
Source: PLoS One. 2014 Feb 21;9(2):e89051. doi: 10.1371/journal.pone.0089051 (PMC3931704; doi:10.1371/journal.pone.0089051)

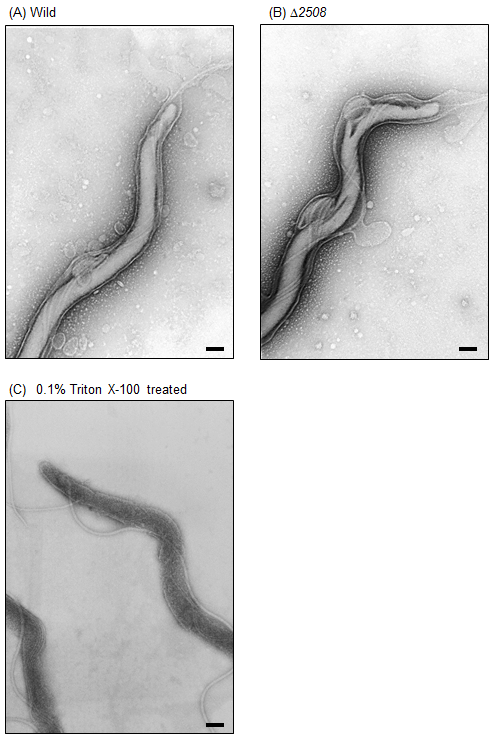

Supplement: Figure S1 — Transmission electron micrographs of T . denticola ATCC 35405 (Wild, A) and tde2508 -deletion mutant (Δ 2508 , B). (C) T. denticola cells treated with 0.1% Triton X-100, showing the disappearance of the surface layer. The bacterial cells were negatively stained with 1% ammonium molybdate, pH 7.0. Bars indicate 100 nm. (TIF) [file pone.0089051.s001.tif]

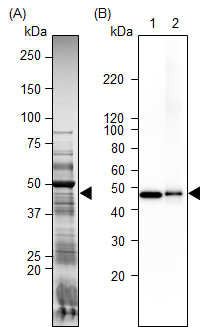

Supplement: Figure S2 — Detection of TDE2508 in surface layer extraction. Cell surface layer was extracted from intact cells of T. denticola ATCC 35405 by suspending in 0.1% Triton X-100. The extraction was subjected to SDS-PAGE with CBB-staining (A) and Western blot analysis with anti-TDE2508 antiserum (B). Lanes 1 and 2 in panel B are the whole cell lysate and the surface layer extract, respectively. The single black arrowheads denote a monomer form of TDE2508. (TIF) [file pone.0089051.s002.tif]

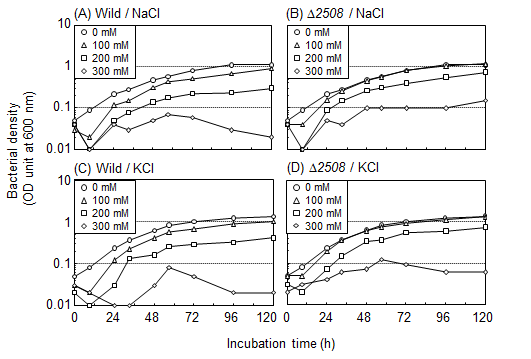

Supplement: Figure S3 — Growth curves of T . denticola ATCC 35405 (Wild, A and C) and tde2508 -deletion mutant (Δ 2508 , B and D). The mGAM-TS medium was supplemented with NaCl (A and B) and KCl (C and D) at 0–300 mM. The strains were anaerobically incubated at 37°C and the optical density (OD) at 600 nm was monitored. The figures representative ones from two independent experiments. (TIF) [file pone.0089051.s003.tif]

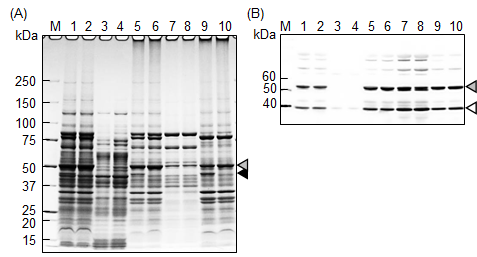

Supplement: Figure S4 — Msp expression. Whole cell lysates of T. denticola ATCC 35405 (Wild) and tde2508-deletion mutant (Δ2508) were fractionated into soluble and envelope fractions. The envelope fractions were further fractionated by differential solubilization in 1% Triton X-100 into soluble and insoluble fractions. The samples were denatured by heating at 100°C for 10 min and subjected to SDS-PAGE with CBB-staining (A) and Western blot analysis with anti-T. denticola whole cell antiserum (B). The odd and even lanes denote Wild and Δ2508, respectively. Lanes 1–2, 3–4, and 5–6 are the whole cell lysate, soluble, and envelope fractions, respectively. Lanes 7–8, and 9–10 are soluble and insoluble fractions in 1% Triton X-100, respectively. The black, grey and white arrowheads denote TDE2508, Msp and TmpC, respectively. M denotes a standard marker. (TIF) [file pone.0089051.s004.tif]

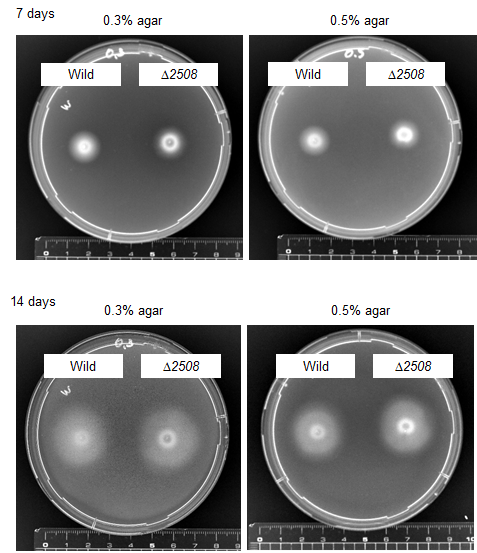

Supplement: Figure S5 — Motility test. T. denticola ATCC 35405 (Wild) and tde2508-deletion mutant (Δ2508) were seeded on mGAM-TS agar plates which were solidified with 0.3% (left) and 0.5% (right) agar. The plates were anaerobically incubated at 37°C, and the turbid plaque was monitored for 2 weeks as an index of bacterial motility. The two strains showed almost the same motility in any concentration of agar over the entire period. Images of the plates at 7 (upper) and 14 (lower) days after the incubation are presented. The numbers in the rulers indicate centimeters. (TIF) [file pone.0089051.s005.tif]

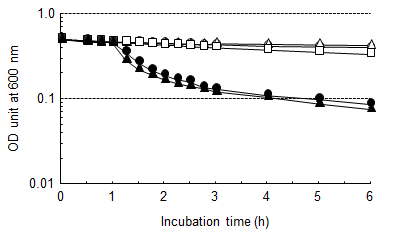

Supplement: Figure S6 — Aggregation assay. For the autoaggregation test, T. denticola ATCC 35405 (open circle) and tde2508-deletion mutant (open triangle) were set in a cuvette, and the optical density at 600 nm (OD600) was monitored. For the coaggregation test, T. denticola ATCC 35405 (closed circle) and tde2508-deletion mutant (closed triangle) were mixed with P. gingivalis, and the OD600 was monitored. The open squares show autoaggregation of P. gingivalis. (TIF) [file pone.0089051.s006.tif]
